# Investigating the composition of fatty acid, tocopherol, and sterol contents and antioxidant capacity of oils from the seeds of *Abelmoschus esculentus* L. growing in Algeria

**DOI:** 10.55730/1300-0152.2726

**Published:** 2024-12-10

**Authors:** Samira NIA, Madjda BENGUECHOUA, Toufik HADJ MAHAMMED, Khatir REGGAGUI, Mohamed YOUSFI

**Affiliations:** 1 Laboratory of Fundamental Sciences, University of Amar Telidji, Laghouat, Algeria; 2 Department of Material Sciences, Faculty of Sciences, University of Amar Telidji, Laghouat, Algeria

**Keywords:** *Abelmoschus esculentus* L, oil, fatty acids, tocopherol content, sterol content, antioxidant activity

## Abstract

**Background/aim:**

Vegetable oils are considered a vital source of energy when glucose cannot be utilized. Therefore, research on new sources of oil that demonstrate nutritional value is necessary. Okra (*Abelmoschus esculentus*), a member of the *Malvaceae* family, is a remarkable botanical specimen due to its various uses; its roots, stem, leaves, fruits, and seeds are all valued for their nutritional, therapeutic, and industrial value. This study aims to demonstrate okra’s fatty acid (FA) composition, tocopherol content, and sterol content and evaluate the antioxidant activity of the oils extracted from okra using three solvents.

**Materials and methods:**

The initial part of this study involved oil extraction using the Soxhlet apparatus with various solvents, followed by the conversion of FAs into methyl esters for analysis via gas chromatography. The second part focused on quantifying tocopherols and sterols using the Emmerie–Engel and Liebermann–Burchard methods, respectively. In the final part, the antioxidant activity of okra oils was measured using the 2,2-diphenyl-1-picrylhydrazyl (DPPH) free radical test.

**Results:**

According to the findings, palmitic acid (28.43%–39.83%) was the primary saturated FA, whereas linoleic acid (19.58%–44.07%) and oleic acid (21.60%–23.59%) were the primary unsaturated FAs. Additionally, the results indicate that the oil extracts from okra seeds contain a substantial amount of tocopherols and a significant quantity of sterols. The results of the DPPH assay show that, while the antioxidant capacity is lower than that of vitamin E, it exceeds that of other vegetable oils.

**Conclusion:**

Our research findings have allowed us to classify this oil as an edible vegetable oil.

## 1. Introduction

The okra plant (*Abelmoschus esculentus* (L.) belongs to the *Malvaceae* family and is grown in tropical and subtropical countries. Its origins can be traced back to Africa. It contains critical elements, such as carbs, proteins, vitamins, and minerals, making it essential for human nutrition (Çiçek, 2024). Okra, known as “bamia” in Arabic, is a popular vegetable crop in Algeria, prized for its distinct flavor and nutritional advantages. The country’s moderate temperature and good soil, particularly in locations such as the Hauts Plateaux and the northern coast, make it suitable for agriculture. Okra is a versatile crop, owing to multiple applications using its flowers, fresh leaves, pods, buds, stems, and seeds. Immature okra fruits used as vegetables can be added to soups, salads, and stews and consumed fresh, dried, fried, or boiled. Okra cooks to a mucilaginous consistency. The extract derived from the fruit is frequently used in various dishes, such as stews and sauces, to improve consistency (Singh et al., 2017). Okra contains several phenolic compounds with crucial biological effects; it is also renowned for having significant antioxidant activity. It provides various potential health benefits for some of the most serious human ailments, including cardiovascular disease, type-2 diabetes, digestive problems, and malignancies (Dantas et al., 2021; Elkhalifa et al., 2021). It is an essential vegetable crop with a wide range of nutritional properties and health advantages (Dantas et al., 2021; Elkhalifa et al., 2021). Okra’s significance goes beyond its fruits; its seeds are also important, as they are a rich source of healthy fats, which play an important role in the body. Some essential fats the body cannot produce and these must be taken through food, mainly vegetable oils. Numerous studies have demonstrated the potential of unsaturated fatty acids (FAs) Omega 3 and 6 in preventing heart disease and their role in the body’s production of beneficial compounds like eicosanoids (Drenjančević and Pitha, 2022). In this study, we focused on the chemical composition of FAs in the lipid extracts of okra seeds, as well as in determining their tocopherol and sterol contents and evaluating their antioxidant activity.

## 2. Materials and methods

### 2.1. Chemicals

Hexane, chloroform, and absolute ethanol were purchased from Fisher Scientific. Sulfuric acid, acetic acid, ferric chloride, vitamin E, and stable 2, 2-diphenyl-1-picrylhydrazyl (DPPH) were obtained from Sigma-Aldrich. All chemicals used were of analytical grade.

### 2.2. Collection of plant material

The okra samples were collected from the Hamda region (town of Laghouat), situated south of Algiers. *Abelmoschus esculentis* (L.) was collected in 2018. The okra samples were air-dried at room temperature for 20 days. The seeds were separated from the fruits and purified.

### 2.3. Lipid extraction

The dried seeds of *Abelmoschus esculentus* L were ground to a fine powder using a coffee mill. The oils were extracted with various solvents (hexane, chloroform, and hexane/chloroform 1/1) using a Soxhlet apparatus for 6 h. The obtained extracts were dehydrated by anhydrous sodium sulfate. After filtration, the solvents were evaporated using a rotary evaporator. The resulting oils were kept at 4 °C.

### 2.4. Fatty acid composition

Gas chromatography was used to determine the FA composition of the oils; for that, the FAs were converted into FA methyl esters according to the following technique: the mixture of 0.2 g of oil and 20 mL of sodium methanolate (0.5%) was refluxed for 30 min; after cooling, 20 mL of water was added; the n-hexane was employed to extract the FA methyl esters, which were washed by water; we dried the extracts over anhydrous sodium sulfate and let it evaporate in a vacuum (Cherbi et al., 2017; Harrat et al., 2018).

### 2.5. Chromatographic analysis of fatty acid methyl esters

The analysis was performed using a Chrompack CP 9002 device equipped with a Flame Ionization Detector, where 1 mL was injected using a split/splitless injector. The capillary column is of the DB23 type with 57% cyanopropyl active phase (length: 37 m; inner diameter: 7.32 mm), and the film thickness is 7.32 mm. The oven was set to a constant temperature of 250 °C. Nitrogen gas was used in the separation as a carrier gas at a flow rate of 1 mL/min while identifying FA methyl esters was carried out by comparing their retention times with those of standard FA methyl esters.

### 2.6. Determination of the total tocopherol content

We estimated the total tocopherol content using the Emmerie–Engel colorimetric method (Emmerie and Engel, 1938). This method is based on the ability of tocopherols to reduce trivalent iron (Fe^3+^) to divalent iron (Fe^2+^). The resulting Fe^2+^ forms an orange-red complex with ortho-phenanthroline, with an absorption peak at a wavelength of 510 nm. Using commercial vitamin E, we prepared solutions of known concentrations in methanol. We took 1 mL of each solution and mixed it with 1 mL of the ortho-phenanthroline reagent, which was made in methanol at a 0.4% concentration, and 0.5 mL of FeCl3 (an ethanolic solution with a 0.12 % concentration). We measured the absorbance at a wavelength of 510 nm after 5 min. We followed the same steps for our lipid extracts.

### 2.7. Determination of the total sterols content

The Liebermann–Burchard test is a widely used method for quantifying sterols. When sterols are treated with Liebermann’s reagent, acetic anhydride and concentrated sulfuric acid react with the 3β-hydroxysteroid, which has a double bond at the 5–6 position, forming a stable compound that produces a bluish-green color. This reaction reaches maximum absorbance at 550 nm. The Berman reagent used in this process consists of 60 mL acetic anhydride, 30 mL acetic acid, and 10 mL sulfuric acid (Barreto, 2005; Harrat et al., 2018). We made several solution dilutions from a chloroform solution of cholesterol with a concentration of 1 g/L to plot a calibration curve that connected the optical density to the concentration. One mL of each diluted solution was added to 2 mL of the Liebermann reagent. The mixture was left for 25 min to let the reagent color fully develop and settle, and we then measured its absorbance at 550 nm. We followed the same procedure for our lipid extracts.

### 2.8. Radical-scavenging activity (DPPH assay)

The method used to determine the antioxidant capacity of our oils was previously described and performed by Yousfi et al. (2009) with some modifications. The assay mixture contained 0.5 mL of serial dilutions of the sample and 250 μM of DPPH prepared in absolute ethanol. This reaction mixture was shaken well and incubated for 30 min in the dark at room temperature. The resulting solution’s absorbance was measured at 517 nm against a blank. The antiradical activity was expressed as IC50 (mg/mL), which is the concentration required to cause 50% initial DPPH inhibition. The inhibitory percentage of DPPH was calculated according to the following formula:


$$I\%\;(inhibition)=\left(\frac{A0-A}{A0}\right)\times 100,$$

where A0 and A are the absorbance values of the control and the tested sample, respectively. Antioxidant of reference vitamin E was used for comparison.

### 2.9. Statistical analysis

Results are reported as means ± SD for three duplicates of each experiment. An AP value of <0.05 indicates significant differences between mean values. The data was statistically analyzed using Excel (Microsoft, Redmond, WA, USA). Strictly linear calibration curves were obtained for all procedures using a variety of calibration standards. Multiple comparison tests were employed to analyze the data.

## 3. Results and discussion

### 3.1. Extraction yields and fatty acid compositions of *Abelmoschus esculentus* seeds

The extraction processes lasted approximately 6 h for each solvent. After evaporating the solvents, we obtained the yields shown in Table 1. The values ranged between 17.55% and 21.04%, with the highest value obtained using the solvent chloroform, followed by the solvent hexane with a value of 18.54% and chloroform/hexane at 17.55%. These results are similar to those of numerous other studies, and when compared to seeds of other *Abelmoschu*s species, the results are similar (Jarret et al., 2011; Dong et al., 2014). Some studies found higher numbers, up to 40%, but these are uncommon (Bawa and Badrie, 2016). Differences in oil yield could be caused by differences in the climate where the seeds are grown, when they are harvested, how they are ripened, and the method used to extract the oil (Benalia et al., 2015). Compared to oilseeds like sunflower and cotton, okra seeds produce a relatively low oil yield. However, given okra’s high productivity and seed-rich fruits, it is a viable alternative for cooking oil production, particularly in response to the growing demand for alternative oil sources (Anwar et al., 2011).

The FA composition of the okra seed oils is presented in Table 1. In the chloroform and hexane/chloroform extracts, the same type of FA was present, while the hexane extract contained only three acids. The main saturated FA components in the chloroform and hexane/chloroform extracts were palmitic acid (C16:0), margolic acid (C17:0), stearic acid (C18:0), and arachidic acid (C22:0); however, the only saturated FA present in the hexane extract was palmitic acid (C16:0). The main unsaturated FAs found in the chloroform and hexane/chloroform extracts were palmitoleic acid (C16:1), oleic acid (C18:1), linoleic acid (C18:2), linolenic acid (C18:3), and gondoic acid (C20:1), while the hexane extract contained only oleic and linoleic acids. The three oils displayed elevated proportions of unsaturated FA compositions in the range of 47.37–67.05%, mostly attributed to elevated levels of polyunsaturated fatty acids (PUFAs) for hexane and chloroform extracts (38.55% and 44.32%, respectively). The oil extract obtained using a mixture of hexane and chloroform exhibited similar percentages of monounsaturated FAs and PUFAs (24.74% and 22.63%, respectively). Conversely, the saturated FAs varied between 21.96% and 38.58%, with palmitic acid being the predominant one (more than 28%). These FA percentages are comparable to those reported in other studies (Awolu, 2014), particularly with chloroform extract. The chloroform and hexane/chloroform extracts included trace levels of the other FAs. Additionally, the UFA/SFA ratios varied from 1.18 to 2.07. The U/S values suggest moderate to acceptable values, which protect the oils against oxidation (Benalia et al., 2015; Harrat et al., 2018). In the hexane/chloroform extract, the monounsaturated FA amounts were higher than the polyunsaturated FA. These findings suggest that okra seed oils could be a valuable source of MUFAs in the diet. Research has shown that a MUFA-rich diet is a beneficial alternative to low-fat diets due to its potential to enhance immune function, lower blood cholesterol levels, reduce the oxidation of LDL, and improve HDL fluidity (Benalia et al., 2015; Harrat et al., 2018). Since the studied oils have a high concentration of linoleic and oleic acids, they are more likely to be liquid than solid and will not readily solidify at room temperature. It has also been suggested that consuming these oils can lower the chance of developing cardiac issues. Okra seed oil appears nutrient-dense since it contains significant concentrations of essential linoleic acid. Because okra seed oil is high in oleic and linoleic acids, it can be used to make margarine, an edible cooking and salad oil. We found that it is relatively lower when comparing the percentage of unsaturated FAs in okra oil to other edible oils, such as cottonseed oil, olive oil, and soybean oil. However, from another perspective, this makes it more oxidation-resistant and better suited for frying than the aforementioned oils (Codex-Alimentarius, 2017).

### 3.2. Quantification of total tocopherols of *Abelmoschus esculentus* seeds

As fat-soluble antioxidants, tocopherols are essential for preventing oxidative stress on various tissues, including the brain and cell membranes. Their antioxidant properties also support the body’s production of prostaglandins by converting arachidonic acid, which helps regulate blood vessel contraction and modulate inflammatory responses. This mechanism reduces platelet aggregation, preventing blood clot formation. Because the body cannot produce tocopherols, consuming tocopherol-rich foods is crucial for preventing various disorders associated with a deficiency, such as cancer, Alzheimer’s disease, and hypertension (Rizvi et al., 2014). Tocopherol contents (Table 2) were determined using a standard curve of vitamin E; in our study, the tocopherol content values were close in the three studied oils. Total tocopherol levels in the okra seed oils were relatively high, in the range of 0.20–0.23 mg/g of “d.w” seeds and 1.13–1.20 mg/g of oil, which likely improved the oil’s oxidative stability during processing and storage. The amount of tocopherols we obtained was lower than values determined in other studies on okra and close to the quantities found in other oils (Table 3), such as cotton oil, which is in the same family as okra, peanuts, and sunflowers; however, the tocopherol amount was less than the quantity found in soybean oil (András et al., 2005; Codex-Alimentarius, 2017).

### 3.3. Quantification of total sterols of *Abelmoschus esculentus* seeds

The food and nutrition, cosmetic, and pharmaceutical industries have recently directed their attention to inexpensive renewable resources abundant in lipid-related substances like phytosterols, which make up a significant portion of the unsaponifiable matter in vegetable oils. Comprehensive information about the identity and quality of the oil under investigation and the identification of oils and blends not identified by their FA profile can be obtained by analyzing sterols (Trabelsi et al., 2011). Sterol contents (Table 2) were determined using a standard curve of commercial cholesterol. Based on the results, we observed that the amount of sterols in the hexane extract (28.99 ± 1.29 mg/g of “d.w”) seeds was very high compared to the chloroform and hexane/chloroform extracts (2.53 ± 0.15 mg/g of “d.w” seeds and 0.99 ± 0.073 mg/g of “d.w” seeds, respectively). The sterol content in okra seed oils was notably high compared to that determined in other studies on okra and significantly surpassed the levels found in most edible oils (Table 3). The existence of other substances with comparable structures, such as vitamin D, beta-carotene, and triterpene alcohols, which react with the Liebermann reagent, may be the cause of this increased sterol content. Nevertheless, okra has a significant amount of plant sterols, making it a valuable source of sterols. These vital nutrients have several health advantages, such as decreasing LDL cholesterol, strengthening the immune system, and protecting the body from some cancers, like colon cancer. They also have antidiabetic qualities that help prevent type 2 diabetes.

### 3.4. Antioxidant activity

The antioxidant activity of okra oils was measured using the DPPH free radical test. DPPH is a stable radical with a maximum absorbance of 515 nm. Antioxidants’ ability to donate hydrogen explains their influence on DPPH radical scavenging. The ability of extracts and pure antioxidants to scavenge DPPH radicals was tested based on their IC50 values, which are defined as the concentration of extract required to reduce the absorbance at 515 nm of DPPH radical solution to half of its initial value. Finally, increased DPPH radical-scavenging activity is associated with a lower IC50 value (Nia et al., 2015). Comparing the DPPH test results shown in Table 4, the antioxidant activity was much lower than that of vitamin E (0.0282 mg/mL), used as a reference antioxidant, because the extracts are oils. Overall, the extract concentrations were close and ranged between 24.52 and 30.62 mg/mL, with the highest efficacy being the hexane extract at a concentration of 24.52 mg/mL, the chloroform extract at 26.53 mg/mL, and the hexane/chloroform extract at 30.62 mg/mL. If we compare the values obtained from the seeds with the values in Table 5, we can observe that they are close, especially the value of the wild mallow, which, like the common mallow, belongs to the same family as okra. One exception is the high value of the oil from jujube leaves because these leaves are aerial parts and contain chloroplasts that release oxygen (energy conversion), and to avoid the oxidation resulting from it, there must be a large amount of antioxidants compared to the other parts. The antioxidant activity in oils is attributed to several compounds, including tocopherols, tocotrienols, and polyphenols. Additionally, okra seeds are rich in polyphenols, including catechins and procyanidins (Ramachandran et al., 2012).

## 4. Conclusion

Finding new oil sources with nutritional qualities is one of the main objectives of the food sector. When glucose is unavailable, lipids, especially vegetable oils, are regarded as a vital energy source for human health. In this study, we examined the oil content, FA profile, tocopherol and sterol levels, and the antioxidant properties of oils of Algerian-grown okra extracted by three different solvents. The oils contained many essential FAs, especially linoleic acid, which is indispensable for human nutrition and linked to health advantages like lowering cholesterol and reducing the risk of heart disease. This research showed that Algerian okra seeds contain detectable amounts of tocopherols, which are crucial for preventing oil oxidation. The amount of sterols was significant, especially in the hexane extract, which had the highest value. Therefore, okra is rich in sterols, making its oil highly nutritional. Okra seeds are a source of oil with a high nutritional value that cannot be ignored, especially since okra has a high yield, is easy to cultivate, and does not require much care. Considering the other uses of the plant mentioned earlier, it would be worthwhile to invest in okra production and use it more frequently in industry and agriculture.

## Figures and Tables

**Table 1:** Extraction yields and fatty acid composition of oils obtained from *Abelmoschus esculentus* seeds.

	Composition %		
solvent	Hexane	chloroform	Hexane/chloroform
Oil %	18.54	21.04	17.55
C16:0	39.83	28.43	33.10
C16:1 (ω7)	/	0.67	0.24
C17:0	/	0.31	0.28
C18:0	/	3.24	4.23
C18:1(ω9)	21.60	21.93	23.59
C18:2 (ω6)	38.55	44.07	19.58
C18:3 (ω3)	/	0.25	3.05
C20:0 (ω9)	/	0.30	0.94
C20:1	/	0.13	0.91
C22:0	/	0.11	1.61
*∑*SFA (%)	39.83	32.39	40.16
*∑*MUFA (%)	21.60	22.73	24.74
*∑*PUFA (%)	38.55	44.32	22.63
*∑*USFA (%)	60.15	67.05	47.37
U/S (%)	1.51	2.07	1.18
**Total identified (%)**	99.98	99.44	87.53

∑SFA: sum of saturated fatty acids; *∑*MUFA: sum of mono-unsaturated fatty acids; *∑*PUFA: sum of polyunsaturated fatty acids; *∑*USFA: sum of unsaturated fatty acids; U/S: ratio of “unsaturated/saturated” fatty acids.

**Table 2:** Tocopherol and sterol contents of *abelmoschus esculentus* seed oils.

oil	Total tocopherol content		Total sterol content	
	(mg/g oil)	(mg/g seeds “d.w”)	(mg/g oil)	(mg/g seeds “d.w”)
Hexane extract	1.20 ± 0.006	0.22 ± 0.0011	156.31 ± 10.50	28.99 ± 1.94
Chloroform extract	1.13 ± 0.016	0.23 ± 0.0035	12.06 ± 3.09	2.53 ± 0.15
Hexane/chloroform extract	1.15 ± 0.024	0.20 ± 0.043	5.68 ± 0.41	0.99 ± 0.073

**Table 3:** Tocopherol and sterol contents in several oils described in the literature.

Oil	Total tocopherol content (mg/g oil)	Total sterol content (mg/g oil)	Reference
*Hibiscus esculentus*	3	12.3	(Andràs,2005)
*Gossypium spp*	0.38–1.20	2.7–6.4	(Codex-Alimentarius, 2017)
*Arachis hypogaea*	0.17–1.30	0.9–2.9	(Codex-Alimentarius, 2017)
*Helianthus annuus*	0.44–1.52	2.4–5	(Codex-Alimentarius, 2017)
*Glycine max L*	0.60–3.37	1.8–4.5	(Codex-Alimentarius, 2017)

**Table 4:** Antioxidant activity (DPPH assay) of *abelmoschus esculentus* oils.

oil	IC50 (mg/mL)
Hexane extract	24.52 ± 0.39
Chloroform extract	26.53 ± 0.82
Hexane/chloroform extract	30.62 ± 0.17
Vitamin E	0.0282 ± 0.00024

**Table 5:** Antioxidant activity (DPPH assay) of several oils described in the literature.

oil	IC50 (mg/mL)	reference
*Malva sylvestris*	28.97	(Tesevic et al., 2012)
*Althaea officinalis*	35.65	(Tesevic et al., 2012)
*Pistacia lentiscus*	3.28–4.51	(Harrat et al., 2018)
*Cucurbita pepo*	17.9–41.7	(Benalia et al., 2015)
